# Chronic pain and use of painkillers, healthcare services and long-term impairment among Syrian refugees: a cross-sectional study

**DOI:** 10.1186/s12889-024-20266-6

**Published:** 2024-10-14

**Authors:** Mari Bakken Standnes, Inger Haukenes, Astrid Lunde, Esperanza Diaz

**Affiliations:** 1 Department of Global Public Health and Primary Care, University of Bergen, Bergen, Norway; 2 Department of Health and Functioning, Western Norway University of Applied Sciences, Bergen, Norway

**Keywords:** Chronic pain, Refugees, Syria, Pain levels, Painkillers, Use of health care, Functional impairment, Trauma, Survey questionnaire

## Abstract

**Background:**

The global increase in forcibly displaced populations highlights the importance of understanding their health needs. Chronic pain is prevalent among refugees, poses significant personal and public health challenges, and complicates their integration into new home countries. Understanding refugees' pain post-migration and how it is being managed is crucial for ensuring adequate and timely interventions and fostering health equity. This paper explores the associations between pain levels and the use of painkillers, healthcare services, and long-term impairment among Syrian refugees with chronic pain, one year after their resettlement in Norway.

**Methods:**

This cross-sectional study is based on survey data collected from 353 quota refugees in 2018–19, one year after resettlement in Norway. The primary outcomes were the use of painkillers, the use of healthcare services, and long-term impairment, according to reported chronic pain levels. Associations between these outcomes and chronic pain levels were studied using Poisson regression, adjusted by sociodemographic variables and trauma experience.

**Results:**

Of the 353 adults included, 52% were women, and the median age was 36 years. A total of 5% reported very mild/mild, 10% moderate, and 12% strong/very strong chronic pain over the last four weeks. Significant associations were found between all chronic pain levels and use of non-prescription painkillers (adjusted relative risks (aRR) (95% CI)); mild (3.1 (2.0–4.7)), moderate (1.8 (1.1–2.8)), strong (1.7 (1.1–2.6)), and prescription painkillers; mild (4.6 (2.2–9.5)), moderate (5.6 (3.2–10.0)), strong (6.7 (3.9–11.3)), compared to those without chronic pain. Use of emergency rooms, specialist care, and hospitalization were significantly associated with strong chronic pain, with aRR (95% CI) of 2.0 (1.2–3.5), 3.9 (2.1–7.0) and 2.4 (1.3–4.4), respectively. Long-term impairment was strongly associated with chronic pain across all pain levels; mild (8.6 (5.6–13.49)), moderate (6.7 (4.3–10.5)) and strong (6.6 (4.3–10.4)).

**Conclusion:**

Despite their young age, more than a quarter of the Syrian refugees in our study reported chronic pain one year after resettlement in Norway. High levels of pain were related to the use of medication, healthcare services, and long-term impairment. Understanding the dynamics of pain among refugees is crucial to ensure adequate and timely management.

**Supplementary Information:**

The online version contains supplementary material available at 10.1186/s12889-024-20266-6.

## Background

By the end of 2023, 6.5 million persons originating from the Syrian Arab Republic had become refugees [1]. Migration impacts refugees' health, and resettlement alters risk profiles, improving some health determinants while worsening others [2]. Refugees may have specific health needs and vulnerabilities compared to host populations, as many come from societies affected by war, conflict and economic crisis. Combined with the effects of a hazardous migration journey, they require sensitive and effective care that addresses both their physical and mental health needs. However, refugees may experience exclusion, stigma and discrimination and restricted access to health care services during their migration trajectory [3].

With respect to pain, some refugee populations might have higher risk of having chronic pain [4–6] also after resettlement [6–8]. Chronic pain is often associated with other health issues and comorbidities [9]. Additionally, there are differences in the treatment of chronic pain among immigrant groups, influenced by individuals’ sociodemographic profiles [10]. Nonetheless, refugees remain underrepresented in pain research [11, 12]. A better understanding of the associations between chronic pain and other health outcomes among refugees resettled in their new country can enhance health by addressing disparities, enabling targeted interventions, and fostering health equity.

Representing a worthy of attention public health challenge in addition to the personal burden, chronic pain affects approximately 30% of the Norwegian population and is the primary reason for visits to general practitioners (GP) [9, 13, 14]. At an individual level, chronic pain is linked to lower quality of life, financial burdens, reduced functionality, social isolation, mental health problems, increased risk of co-morbidities, mortality, and suicide [9]. Trauma experience has also been widely linked to chronic pain, with both physical and psychological distress contributing to the long-term development and persistence of pain symptoms [15, 16]. In the majority population, low socioeconomic status is associated with pain and pain-related disability, and more frequent use of strong painkillers [14, 17, 18], underlining the complex relationship between social determinants and pain [19]. Societally, chronic pain contributes to numerous disability benefits and long-term sick leaves [9, 14].

According to the CHART study (Changing Health and Health Care Needs Along the Syrian Refugees’ Trajectories to Norway) [20], and the REFUGE study (Health and quality of life among Syrians in Norway) [21] in Norway and other international studies, most Syrian refugees experience similar or improved health outcomes after resettling in high-income countries, compared to pre-resettlement [7, 22, 23]. However, 25% to 43% of Syrian refugees experience chronic pain after resettlement [7, 8, 24] (compared to 30% reporting chronic pain *pre*-resettlement [7]), with those affected reporting comorbidities [22, 23] and poorer health [8, 24]. Further, chronic pain is often linked to mental health conditions such as anxiety, depression, and PTSD [8, 23–26].

Potential explanations for chronic pain among refugees after resettlement include factors related to the different spatial and temporal stages of the migration process; yet significant knowledge gaps persist. Aversive pre-migration experiences, such as traumatic events, have been shown to be associated with the onset of chronic pain [27–29]. Furthermore, evidence suggests that although poor mental health experienced during transit is not directly associated with chronic pain at the time, it can predict its onset after resettlement, even among refugees with no prior history of pain [23]. Economic hardship at the individual level in transit settings, along with the migration pathway itself, have also been linked to chronic pain following resettlement [5, 23]. Challenges after resettlement further exacerbate this issue, as refugees may face discrimination, anxiety, depression, ongoing economic struggles, inadequate safety, limited access to information, substandard living conditions, and barriers to healthcare—factors all shown to be associated with chronic pain [5, 23, 28, 30]. Additionally, poor mental health in the post-resettlement phase has consistently been linked to chronic pain, highlighting the complex interplay between psychological distress and physical health outcomes among refugee populations [23]. Furthermore, recent studies emphasize the greater importance of factors during the migration trajectory, particularly after resettlement, in understanding chronic pain in refugees [5, 6, 23, 31].

Refugees in Norway have basically the same healthcare rights as the general population, including GP access, and they are offered a health assessment within three months after resettlement [32]. Despite universal coverage and minimal out-of-pocket expenses, health inequities are increasing [33], and unmet health needs have been suggested within the Syrian refugee population in Norway [30]. In other high-income countries, such unmet needs range from 27 to 49% within Syrian populations [22, 24].

Poor access to healthcare among Syrian refugees has been found to be associated with chronic pain [28]. One year after arrival to Norway, the CHART study revealed that the use of GP and emergency care (ER) among Syrian refugees increased compared to the use in transit in Lebanon, while use of specialist healthcare dropped, and hospitalization rates remained the same [34]. Factors such as age, health literacy, educational level, social support, self-rated health, and quality of life have been associated with healthcare services use among Syrian refugees’ post-resettlement [34]. However, limited knowledge exists on healthcare service use among refugee populations suffering from chronic pain.

Chronic pain is often associated with an increased use of painkillers [35] and a higher risk of functional impairment, affecting daily activities and quality of life [9]. Ten to 17% of Syrian refugees in Norway use painkillers daily [30, 36], with higher use among women [30]. According to CHART, daily painkiller use among those with chronic pain increased from 21% during transit to 29% after resettlement. Although there has been found evidence of inadequate treatment among Syrian refugees reporting non-communicable diseases [30], less is known about the association between levels of pain and use of painkillers among refugees after resettlement.

Among Syrian refugees residing in Norway, the prevalence of functional impairment ranges between 28 and 35% [7, 8]. Findings from the REFUGE study indicate associations between severe chronic pain and functional impairment [8]. The intricate and bidirectional relationship between chronic pain and impairment [14] raises concerns since functional levels among Syrian refugees' do not seem to improve after resettlement [7]. This can hinder integration [30], limit socioeconomic status, employment, societal integration, and overall health improvements [14, 37].

The interplay between levels of chronic pain, painkiller use, healthcare use, and long-term impairment is complex. This study seeks to provide knowledge that can guide policy makers and practices in their planning of healthcare provision for refugees with chronic pain, ant thereby contribute to adequate treatment for the individual, and equity in healthcare for the entire population. In this article we aim to 1) describe different chronic pain levels among newly arrived Syrian refugees in Norway and 2) examine the associations between levels of chronic pain and use of health care services, use of painkillers and long-term impairment.

## Methods

### Study design and participants

This paper presents observational, cross-sectional secondary analyses from the CHART project, which is described in depth elsewhere [20, 23, 30]. One-year post-resettlement in Norway (2018–2019), Arabic-speaking study staff invited the CHART cohort to answer follow-up data through a structured telephone interview. The inclusion criteria for the CHART project at baseline were adult Syrian refugees of 16 years and above under the UNHCRs (United Nations High Commissioner for Refugees) international protection mandate living in Lebanon in 2017–18 admitted for resettlement in Norway [38]. Exclusion criteria were unaccompanied refugee minors between 16 to 18 years and persons with severe mental disorders [7, 39]. A total of 506 participants were recruited at baseline, with an overall response rate of 98%. Of these, 464 (92%) were confirmed resettled in Norway after one year, of whom 353 (76%) completed the follow-up questionnaire. Thus, the final study sample was *N* = 353, of which 138 (53%) were women.

### Exposure variable

We used self-reported chronic pain as the exposure in this study, measured using questions previously validated for population studies [40]. Participants were first asked if they had chronic pain, defined as experiencing physical pain for the last six months. If the answer was positive, they were further asked to describe how strong their physical pain had been during the previous four weeks, on a 6-point Likert scale ranging from *no pain* to *very strong*. Those with missing values on the Likert scale were assumed to represent no pain (the same sample that responded *no* to the chronic pain question) and merged into the *no-pain category*. V*ery mild* and *mild* pain were merged into a second category, *moderate* pain was kept as a third category, and *strong* and *very strong* pain were merged into a fourth category [40].

### Outcome variables

The outcome variables were self-reported use of painkillers, self-reported use of healthcare services such as general practitioner (GP), emergency room services (ER), outpatient health services, use of specialist, hospitalization, and self-reported level of long-term impairment.

The use of painkillers (non-prescription and/or prescription) was assessed by questions from the Oslo Health Study (HUBRO) [41], and participants were asked to report the frequency of their use (daily, weekly, less than weekly or not at all) for the last four weeks. Variables were dichotomized by merging *daily, weekly, less than weekly* as yes/1 and *not at all* as no/0.

Use of health care and functional impairment were assessed using questions from The Nord-Trøndelag Health Study (HUNT) [42]. The participants were asked the multiple-response question: ‘During the last 12 months, have you visited any of the following: General practitioner, emergency room services’, another specialist outside the hospital, consultation with doctor without being admitted, and ‘Have you been admitted to the hospital the last 12 months? (yes/no). We combined the responses *another specialist outside the hospital* and *consultation with doctor without being admitted*, given the similarities in outpatient and specialist health care [34].

Long-term impairment was defined as mental or somatic health problems or injuries impairing daily life and lasting at least one year [43]. If the participants reported a long-term impairment, they were asked to specify their impairment(s) (motor ability, vision, hearing, physical illness and/or mental health problems) and describe each impairment on a Likert scale (slight/moderate/severe). Due to few observations in some categories and for simplicity, values on this scale were merged into *yes*/1, and missing values into *no*/0 to create dichotomous outcomes.

### Adjustment variables

Sociodemographic variables collected through the questionnaire included gender, age, years of education, marital status, number of children, and occupational status. These variables were incorporated to provide a descriptive overview of the study population. The introduction programme, which was one of the possible answers to the question regarding occupational status, is a training initiative designed to prepare refugees for integration into Norwegian society and the labor market [44]. It is compulsory for all refugees aged 18 to 55 who arrive in Norway and typically lasts for two years. Participation in the introduction programme is considered crucial for successful societal integration and was therefore considered useful to include in the descriptive data. However, only *gender*, *age*, and *years of education* were utilized as sociodemographic adjustment variables in the subsequent statistical analyses as empirical evidence have demonstrated a possible connection between these variables and the outcome measures [6, 8, 9, 14, 30, 34, 45].

Given the potential relationship between chronic pain and trauma [28], the Single General Trauma Item [46] was included to identify previous exposure to traumatic events, assessing exposure to stressful events of exceptionally threatening or catastrophic nature, which could cause pervasive distress. Examples include assault, witnessing harm or death. Participants were asked to indicate whether they had experienced such events (yes/no). Age and education were used as continuous variables, and gender and trauma as categorical variables.

### Statistical analysis

We used crude prevalence proportions, median and interquartile range (IQR) to describe the exposure, adjustment and outcome variables, both overall and stratified by levels of chronic pain. Associations between chronic pain and various binary outcomes were estimated using Poisson regression (log-link) with robust standard errors, within a Generalized Linear Model framework. This model was selected because binomial regression (log-link) failed to converge [47]. Robust standard errors were used to correct for potential overestimation of standard errors and confidence intervals in Poisson regression for binary data. Associations were calculated as incidence rate ratios and reported as relative risks (RR) with 95% confidence intervals (CIs). RRs was determined unadjusted (Model 1), adjusted for gender, age, and education (Model 2), and additionally adjusted for reported previous trauma (Model 3).

Potential confounding variables for the association analyses were identified based on previous literature [7, 23] and visualized through a Directed Acyclic Graph (DAG) (Figure A1—Additional file 1) using the DAGitty software [48]. DAGs help visually represent the relationships between variables and identifying potential casual pathways, and also clarify which variables may confound the relationship between exposure and outcome [48]. Different confounding adjustments variables were considered as well as an interaction between previous trauma and chronic pain, as previous studies have indicated a strong association between pain and trauma [4, 27]. Initially, the interaction between previous trauma and chronic pain was investigated by stratifying for previous trauma to discern any differential associations between chronic pain and the outcomes. However, no convincing evidence of an interaction between trauma and chronic pain was found for any of the studied outcomes. Consequently, trauma was included as an adjustment variable in the final adjustment model.

**Fig. 1:**
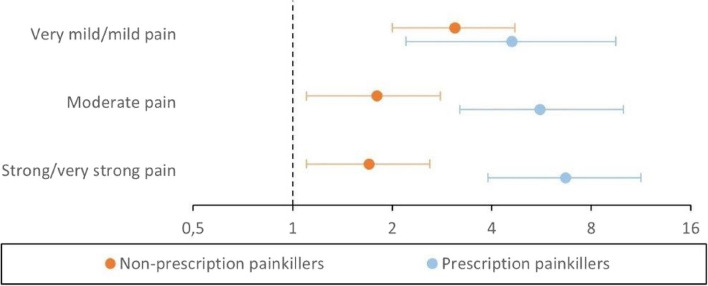
Associations between levels of chronic pain and use of painkillers. Log-scaled coefficient plot illustrating the fully adjusted associations (Table A1a, Model 3) between pain levels last four weeks and use painkillers last four weeks shown by aRR (dot) and 95% CI (bar). Note that a value of 1 indicates the reference group without pain

Missing values were handled through stepwise deletions. Data were analyzed using Stata/SE software, version 17.0, StataCorp LLC, Texas, USA.

### Ethics

The CHART- project was approved by the Regional Committee for Medical & Health Research Ethics of South East Norway (reference 2017/377) and is conducted in accordance with the Declaration of Helsinki. Informed consent was obtained from all participants both prior to the baseline study (Lebanon 2017–2018) and again orally at the follow-up study [23].

## Results

### Characteristics of the study population by levels of chronic pain

Overall, 27% reported some level of pain; 5% of the participants reported very mild/mild, 10% moderate, and 12% strong/very strong chronic pain (Table 1).

**Table 1:** Sociodemographic variables, trauma experience, and outcome variables by chronic pain levels (*N* = 353)

	**Reported chronic pain levels during the last four weeks**^**a**^									
	**No pain** (*n* = 257)		**Very mild/mild** (*n* = 19)		**Moderate** (*n* = 35)		**Strong/very strong** (*n* = 42)		**Total** (*n* = 353)	
**SOCIODEMOGRAPHIC VARIABLES**										
**Gender** (women), n (%)	138	(54)	6	(32)	16	(46)	21	(53)	181	(52)
**Age** in years, median (IQR)	35	(24–41)	40	(34–44)	40	(34–48)	42	(37–47)	36	(29–43)
**Marital status,** n (%)										
Married	180	(71)	17	(89)	30	(88)	33	(79)	260	(74)
Other^b^	74	(29)	2	(11)	4	(12)	9	(21)	89	(26)
**Children**, n (%)										
1–2	39	(20)	1	(6)	6	(19)	7	(18)	53	(19)
3–4	107	(56)	9	(50)	19	(59)	17	(44)	152	(54)
5 or more	46	(24)	8	(44)	7	(22)	15	(38)	76	(27)
**Education** in years, median (IQR)	8	(6–10)	7	(6–9)	7	(5–9)	7	(5–9)	8	(6–9)
**Occupational status Norway**, n (%)										
Student/Introduction programme	236	(94)	17	(89)	30	(86)	34	(81)	317	(91)
Other^c^	15	(6)	2	(11)	5	(14)	8	(19)	30	(9)
**TRAUMA EXPERIENCE,** n (%)										
Exposure to traumatic event(s)^d^	95	(37)	11	(58)	24	(71)	22	(54)	152	(43)
**OUTCOME VARIABLES**^e^**,** n (%)										
**Use of painkillers**^f^										
Non-prescription	63	(25)	14	(74)	15	(43)	20	(48)	112	(32)
Prescription	21	(8)	7	(37)	17	(49)	24	(57)	69	(20)
**Use of health care services**^g^										
General practitioner	213	(83)	17	(89)	32	(91)	38	(90)	300	(85)
Specialist/Outpatient^h^	26	(10)	2	(11)	10	(29)	17	(40)	55	(16)
Emergency room services	36	(14)	5	(26)	5	(14)	17	(40)	63	(18)
Been admitted to hospital	32	(12)	2	(11)	7	(20)	16	(39)	57	(16)
**Impairment**										
Long-term impairment^i^	26	(10)	17	(89)	27	(77)	30	(71)	100	(28)
Motor ability impairment	16	(6)	15	(79)	21	(60)	29	(69)	81	(23)
Vision impairment	8	(3)	1	(5)	3	(9)	12	(29)	24	(7)
Hearing impairment	6	(2)	2	(11)	3	(9)	7	(17)	18	(5)
Impairment due to physical illness	9	(4)	8	(42)	13	(37)	18	(43)	48	(14)
Impairment due to mental illness	3	(1)	2	(11)	1	(3)	11	(26)	17	(5)

^a^Participants were asked if they had “physical pain now that has lasted for more than 6 months” and those who responded *yes*, were further asked to describe how strong their physical pain had been during the last four weeks on a scale from “No pain” to “Very strong”. Those who responded *no* to the first question is shown as “No pain” (*n* = 253) merged with those who responded *no pain* (*n* = 4) on pain levels

^b^Single, divorced, separated, widowed or other

^c^Employed for wages, out of work, homemaker, retired, unable to work

^d^Measured by the Single General Trauma Item, and defined as exposure to a stressful event or situation (either short or long lasting) of exceptional threatening or catastrophic nature, such as being assaulted, or witnessing other people being hurt or killed

^e^The variables presented below include data from participants (n) who responded *yes* to the related question, and the corresponding percent

^f^Use of painkillers during the last *four weeks*

^g^Use of health care services during the last *12 months*

^h^Visited another specialist outside the hospital and/or consultation with doctor without being admitted (merged)

^i^Defined as long-term (at least one year) illness or injury of a physical or psychological nature that impairs daily life

The gender distribution was similar for different levels of chronic pain, with approximately 50% women in each group, except for very mild/mild pain (32%women) (Table 1). Median age increased with increasing level of chronic pain. Compared to those who reported no chronic pain, participants reporting strong/very strong chronic pain were more likely to be married, and having 5 or more children, and less likely to having more than 9 years of education and being in the introduction programme (student) (Table 1). Participants reporting moderate chronic pain had the highest prevalence of self-reported trauma experiences, 71% vs. 37% (no pain).

### Levels of chronic pain and use of painkillers

During the four weeks before the interview, almost one-third of the sample had used non-prescription painkillers, while one-fifth had used prescription painkillers (Table 1). The use of non-prescription painkillers was most prevalent among those with mild chronic pain (74%), while prescription painkillers increased with reported pain level. Still, 25% and 8% of those who reported *no chronic pain* also used non-prescription painkillers and prescription painkillers, respectively.

Participants reporting chronic pain, regardless of pain level, were significantly more likely to use non-prescription painkillers compared to the reference group with no chronic pain. The adjusted relative risks (aRR) for non-prescription painkiller use were 3.1 (95% CI: 2.0–4.7) for mild pain, 1.8 (1.1–2.8) for moderate pain, and 1.7 (1.1–2.6) for strong pain (Fig. 1 and Table A1a, Model 3 – see Additional file 2).

The likelihood of using prescription painkillers increased with the severity of chronic pain, with aRRs of 4.6 (2.2–9.5) for mild pain, 5.6 (3.2–10.0) for moderate pain, and 6.7 (3.9–11.3) for strong pain (Fig. 1 and Table A1a, Model 3).

The fully adjusted models (Model 3) for the outcome variables related to the use of painkillers showed only slight attenuation compared to the unadjusted and partially adjusted models (Table A1a).

### Levels of chronic pain and use of healthcare services

**Fig. 2:**
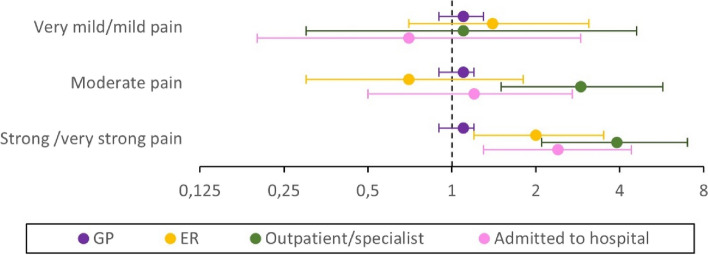
Associations between levels of chronic pain and use of health care services. Log-scaled coefficient plot illustrating the fully adjusted associations (Table A1b, Model 3) between pain levels last four weeks and use of general practitioner (GP), emergency room services (ER), specialist health care and hospitalization during the last 12 months shown by aRR (dot) and 95% CI (bar). Note that a value of 1 indicates the reference group without pain

Most participants (*n* = 300, 85%) had visited a GP, regardless of reporting chronic pain (Table 1). There was limited evidence of an association between chronic pain and GP visits across different levels of chronic pain in both unadjusted and adjusted models, with an adjusted relative risk (aRR) of 1.1 (95% CI: 0.9–1.2 or 1.3) for all levels of pain (Table A1b, Model 3).Participants reporting strong/very strong chronic pain were twice as likely (aRR 2.0 (1.2–3.5)) to have used emergency room service (ER) compared to those without chronic pain. The association was slightly attenuated after adjustment, with the unadjusted RR of 2.9 (1.8–4.6) decreasing to a fully adjusted aRR of 2.0 (1.2–3.5) (Table A1b). No significant associations were observed between mild or moderate chronic pain and use of ER compared to no chronic pain (Fig. 2 and Table A1b).

In total, 16% of the participants had used specialist and/or outpatient care (Table 1). Significant differences in the use of such services were observed among those reporting moderate chronic pain (aRR 2.9 (1.5–5.7)) and strong chronic pain (aRR 3.9 (2.1–7.0)) compared to those reporting no chronic pain (Fig. 2 and Table A1b, Model 3).

Participants with strong chronic pain were twice as likely (aRR 2.4 (1.3–4.4)) to have been admitted to hospital compared to those without chronic pain (Table A1b, Model 3). No significant associations were found between mild or moderate chronic pain and hospital admission (Fig. 2 and Table A1b).

Consistent with the previous analysis, the estimates for healthcare services remained largely stable across all three models, with only minor adjustments (Table A1b).

### Levels of chronic pain and impairment

**Fig. 3:**
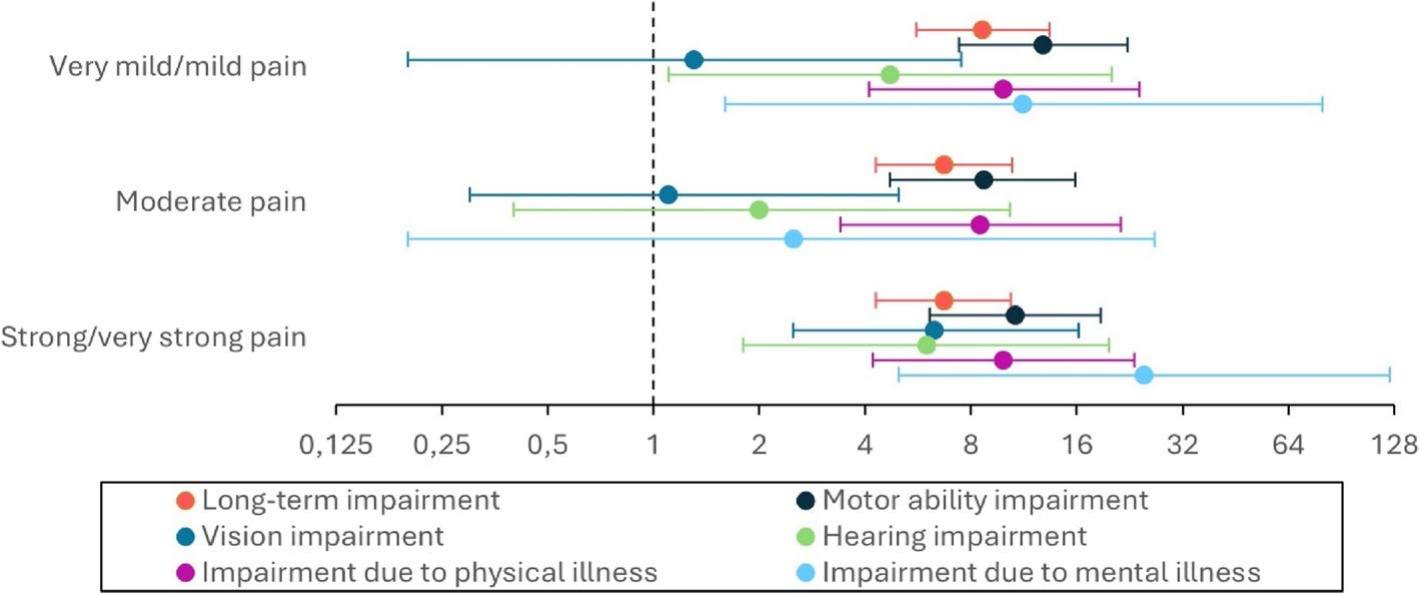
Associations between levels of chronic pain and impairment. Log-scaled coefficient plot illustrating the fully adjusted associations (Table A1c, Model 3) between pain levels last four weeks and long-term impairment (lasting > 12 months) and type of impairment, shown by aRR (dot) and 95% CI (bar). Note that a value of 1 indicates the reference group without pain

Almost one-third (28%) of all participants reported long-term impairment lasting at least 12 months, with the majority among those reporting chronic pain (Table 1). Participants with chronic pain reported long-term impairment significantly more often than those without chronic pain, with adjusted relative risks (aRR (95% CI)) of 8.6 (5.6–13.4) for mild pain, 6.7 (4.3–10.5) for moderate pain, and 6.7 (4.3–10.4) for strong pain (Fig. 3 and Table A1c). Introducing the trauma variable in Model 3 (Table A1c), reduced the fully adjusted estimates for long-term impairment from an unadjusted RR of 7.6 (5.1–11.5) to an aRR of 6.7 (4.3–10.4) for those with moderate pain.

We also found associations between chronic pain and motor ability impairment, with adjusted relative risks (aRR) of 12.8 (7.4–22.3), 8.7 (4.7–15.8) and 10.7 (6.1–18.7) for mild, moderate and strong pain respectively (Fig. 3 and Table A1c). Like previous patterns, adjustment for trauma in Model 3 (Table A1c) reduced the estimates for moderate pain, with the unadjusted RR decreasing from 9.6 (5.6–16.7) to an aRR of 8.7 (4.7–15.8).

Those with strong pain were about six times more likely to report vision impairment compared to those without chronic pain (Fig. 3 and Table A1c). This was the only significant association after adjustments. Initially, the association was stronger, decreasing from an unadjusted RR of 9.2 (4.0–21.1) to 7.2 (2.7–19.0) with sociodemographic adjustments (Model 2), and further to a fully adjusted aRR of 6.3 (2.5–16.5) after including the trauma variable (Model 3).

For hearing impairment, we observed associations with both mild and strong pain, with adjusted relative risks (aRR) of 4.7 (1.1–20.1) and 6.0 (1.8–19.7), respectively. Adjusting for sociodemographic variables (Model 2, Table A1c) reduced the strong pain estimate from an unadjusted RR of 7.1 (2.5–20.2) to 5.7 (1.6–20.8). Further adjustment with the trauma variable (Model 3) kept the estimate at 6.0 (1.8–19.7).

Impairment due to physical illness was high across all pain levels, with adjusted relative risks (aRR) of 9.9 (4.1–24.1), 8.5 (3.4–21.3), and 9.9 (4.2–23.3) for mild, moderate, and strong pain, respectively (Table A1c, Model 3). Sociodemographic adjustments (Model 2) slightly reduced estimates for strong pain. Trauma adjustment further decreased estimates across all pain groups (Table A1c, Model 3).

Finally, we observed an association between pain and mental illness for very mild and strong pain, with adjusted relative risks (aRR) of 11.2 (1.6–79.8) and 24.9 (5.0–123.9), respectively. The wide confidence intervals for these associations limit the interpretability of the findings.

## Discussion

Among newly arrived refugees from Syria to Norway, in a non-clinical setting with nearly one-third (27%) reporting chronic pain, this study provides valuable insights into the associations between levels of chronic pain and use of painkillers, healthcare services, and long-term impairment. Although most refugees in our study did not report having chronic pain, those who did experienced levels that were high for their age compared to the general population [40]. Experiencing chronic pain was associated with a higher likelihood of both non-prescription and prescription painkiller use, as well as increased use of certain healthcare services, such as ER visits, specialist care, and hospitalizations, alongside long-term impairment. All levels of chronic pain reported in the four weeks prior to our interview were significantly linked with an increased likelihood of long-term impairment.

In comparison with the Norwegian HUNT pain study [40], a general population study that used identical validated questions about chronic pain in individuals aged 20 years and above in the general population, our data revealed notable differences. In the HUNT pain study [40], 18% reported mild pain (vs. 5% in our population), 24% reported moderate pain (vs. 10% in our population), and 2% reported severe pain (vs. 12% in our population). However, our study involved a younger population (median age 36 years) compared to the HUNT pain study (median age 54 years), suggesting a higher pain level for their age in our Syrian population. This underscores the need to support refugee subgroups with unique health needs that differ from the wider population [7]. The real differences between the groups are likely to be even larger, given that the Syrian population in Norway is younger than the general population [49, 50].

To our knowledge, only the CHART study and the REFUGE study have previously examined pain levels among Syrian refugees in Norway. The REFUGE study found that 43% experienced severe chronic pain, while 37% reported some chronic pain, and 20% reported no pain. However, the REFUGE study used 10 items on pain lasting for ≥ 3 consecutive months last year, making direct comparisons with our findings challenging. Additionally, the REFUGE study had only a 10% response rate, which might imply a more selected population [8].

In our study, significant associations were observed between all levels of chronic pain levels and painkiller usage (Fig. 1), with the most pronounced associations for prescription painkillers. Participants with mild chronic pain had the highest use of non-prescription painkillers, consistent with findings from previous studies [35]. The higher cost of non-prescription painkillers, such as Paracetamol or NSAIDs, in Norway may explain why individuals with severe pain might be prescribed these medications when treated, as they are much more expensive to purchase without a prescription.

A previous study in the same CHART cohort described a decrease in daily painkiller use after resettlement compared to the transit phase among those not reporting chronic pain [23]. Nevertheless, one year later, a relatively high proportion of participants without chronic pain reported using painkillers, with 25% and 8% using non-prescription and prescription painkillers, respectively, during the last four weeks. Whether these findings indicate unmet healthcare needs remains uncertain and should be further studied.

A total of 85% of our sample had visited a GP in the last year, with no significant differences found between those reporting pain and those without pain (Fig. 2). For comparison, 75% of the general population reported using a GP in the last 12 months [51]. The high utilization could be attributed to initial health assessments often conducted by a GP [34], especially since this cohort reported relatively good health after arrival [30, 39].

The significant associations between the use of ER services, specialist care, and hospitalization among those with strong pain (Fig. 2) might indicate effective pain management. However, the previously described relationship between chronic pain and comorbidities should also be considered [8, 9, 23]. A total of 16% of the refugees, regardless of pain level, had been admitted to the hospital during the past year, compared to 11% of the general population of the same age [51].

Almost one-third of the refugees reported long-term impairment, regardless of chronic pain (Table 1). In comparison, 17% of the general Norwegian population aged 25–44 years reported long-term impairment, consistent with data for the 15–66 age group from Statistics Norway [43]. Among those with pain, 77% had long-term impairment, which is a high prevalence and concerning. To address this, future research should focus on developing targeted interventions and policies to manage the complex, bidirectional effects of chronic pain and impairment on refugees’ daily lives.

Our regression analyses showed that sociodemographic factors like gender, age, and education had limited explanatory value for the associations between chronic pain and the outcome variables, with minimal changes in estimates (Table A1a, A1b, A1c). These factors generally do not account for the observed associations, suggesting that migration experience may have a stronger influence on pain medication use, healthcare services, and long-term impairment than individual variations in sociodemographic characteristics. Thus, migration status appears to be the most important factor.

In line with our findings, trauma experience has been associated with chronic pain among refugees [27, 30], and our study’s adjustment for trauma slightly attenuated some associations between chronic pain and impairment (Table A1c, Model 3). However, the binary yes/no measure of trauma may not fully capture its complexity, potential leading to residual confounding [52].

Both refugees and physicians may lack knowledge about trauma’s complex impact on health [53], which can result in physicians rarely inquiring about trauma or refugees not disclosing their trauma histories. Given the links between chronic pain and trauma, it is crucial for physicians to inquire about trauma and mental health to ensure appropriate referral to psychosocial services and comprehensive care. Without this, there could be unmet healthcare needs and increased self-medication for symptoms beyond pain. Previous studies suggest a connection between non-prescription painkiller use and psychological distress, such as depression and anxiety [54], which is commonly observed in the general population [35]. To our knowledge, this relationship has not been studied among refugees in high-income countries and warrants further exploration.

Our analysis showed strong associations between chronic pain and painkiller usage, which persisted even after adjusting for trauma experience (Table A1a, Model 3). Nonetheless, biases such as underreporting pain or misreporting could explain painkiller use among those without chronic pain. Introducing the trauma variable led to reductions in risk estimates for various impairments, though not always statistically significant, and wide confident intervals, especially for mental illness, warrant caution. This underscores the need to consider trauma in understanding chronic pain and related impairments, highlighting the interplay between migration experiences and health outcomes.

However, including trauma variables in refugee health research risks reinforcing the “traumatized refugee” narrative, which can contribute to overgeneralization and stigmatization of refugees as a homogenous group with a singular, traumatic identity [55]. Given that chronic pain is known to be associated with trauma in the general population [14–16], the adjusting for trauma was deemed pertinent. It is also important to address the potential for re-traumatization when incorporating such variables in questionnaires. To mitigate this risk, trained health personnel were present during the initial phase of the CHART study, as described in previously published papers [30, 39].

Our study also identified that refugees with severe pain were less likely to participate in the Introduction programme (81%) compared to those without pain (94%) (Table 1), despite the right to a customized program for those with health challenges [44]. This finding, in line with previous research relating impairment to post-migration unemployment [37] and work disability [56], suggests a need for improved adaptation of the Introduction programme for refugees suffering from chronic pain and functional impairments to ensure better integration.

Given that chronic pain is a significant global public health issue, our findings may provide valuable insights that could be relevant to other refugee populations.

### Strengths and weaknesses

The main strength of our study is the high response rate, enhancing the generalizability and minimizing the risk of response bias. Also, the data collected relied predominantly on validated survey items and followed validated translation and adaptation principles. However, there are limitations to consider. We assessed chronic pain levels only for the last four weeks before the interview, capturing a relatively short-term snapshot of participants' chronic pain experiences. Consequently, the term 'chronic pain levels' in our context may somewhat misrepresent the broader and enduring nature typically associated with chronic pain conditions. Future studies incorporating questions covering a more extended observation period could offer a more nuanced understanding of the chronic pain experiences over time*.* Further, while our dataset shows high general practitioner (GP) usage and a significant association between strong pain and healthcare service use, it doesn't capture the frequency of consultations per refugee. Additionally, our dataset does not include information on the usage of psychiatric health services, creating a gap in understanding the complex relationship between pain, trauma, and mental illness. Lastly, our method of merging variables to measure the frequent use of painkillers does not provide estimates for daily use, complicating comparisons to other studies.

## Conclusion

Although most Syrian refugees do not report chronic pain one year after resettlement, those who do experience high levels of chronic pain for their age. The association between chronic pain and long-term impairment may place these refugees in vulnerable situations, potentially affecting their integration into society. Policymakers must ensure timely and appropriate diagnosis and treatment. Future research should also examine how chronic pain and overall health status of refugees change over time following resettlement.

## Supplementary Information

Additional file 1.

Additional file 2.

## Data Availability

The datasets generated and/or analyzed during the current study are not publicly available due to data protection regulations in Norway but are available from the corresponding author on reasonable request.

## Abbreviations

aRR: Adjusted relative risks
GP: General practitioner
ER: Emergency room care
CHART: Changing Health and Health Care Needs Along the Syrian Refugees’ Trajectories to Norway
REFUGE: Health and quality of life among Syrians in Norway
UNHCR: United Nations High Commissioner for Refugees
HUBRO: The Oslo Health Study
HUNT: The Nord-Trøndelag health Study
