# Supplementary figures and images for: Chronic pain and use of painkillers, healthcare services and long-term impairment among Syrian refugees: a cross-sectional study
Source: BMC Public Health. 2024 Oct 14;24:2815. doi: 10.1186/s12889-024-20266-6 (PMC11472554; doi:10.1186/s12889-024-20266-6)

**Additional file 1:**

Figure A1 -Directed Acyclic Graph (DAG)


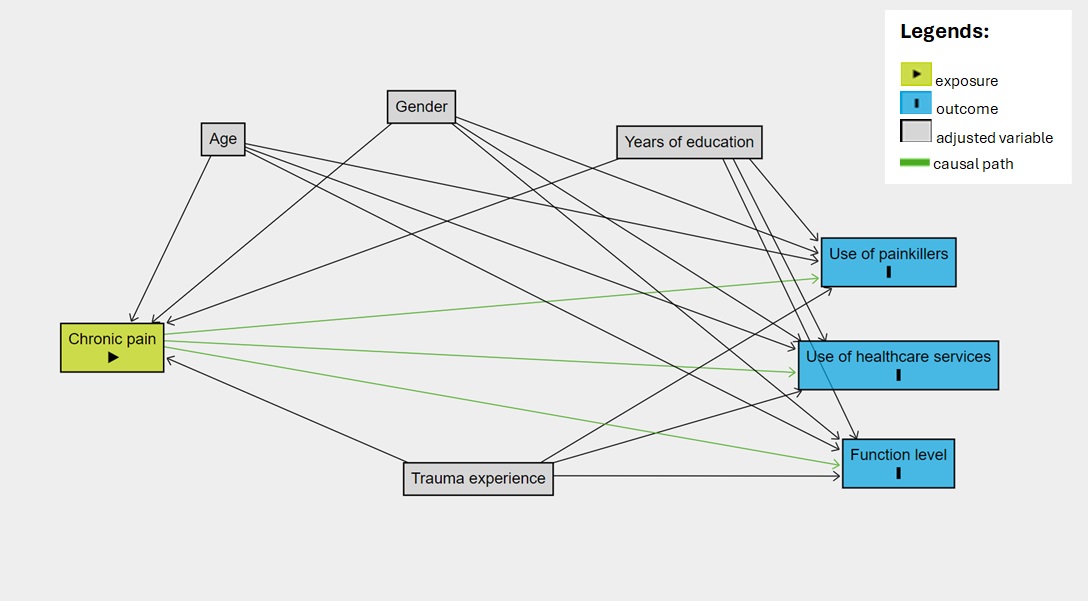

Supplement: Supplementary file 1 — Additional file 1. [file 12889_2024_20266_MOESM1_ESM.docx]
