# Supplementary material for: Chronic pain and use of painkillers, healthcare services and long-term impairment among Syrian refugees: a cross-sectional study
Source: BMC Public Health. 2024 Oct 14;24:2815. doi: 10.1186/s12889-024-20266-6 (PMC11472554; doi:10.1186/s12889-024-20266-6)
Supplement: Supplementary file 2 — Additional file 2. [file 12889_2024_20266_MOESM2_ESM.docx]

**Additional file 2:**

Table A1a, A1b & A1c: Regression analysis with adjustment models

| Table A1a: Associations (relative risk) between reported levels of chronic pain during the last four weeks and use of non-prescription painkillers and prescription painkillers during the last 4 weeks (N=353) | | | | |
| --- | --- | --- | --- | --- |
|  |  |  | | |
|  |  |  | **RR (95% CI)** |  |
| Chronic pain levels |  | **Model 1^1^** | **Model 2^2^** | **Model 3^3^** |
|  |  | **Non-prescription painkillers (n=112)** | | |
| No pain (reference) |  | 1 | 1 | 1 |
| Very mild/mild |  | 3.0 (2.1 - 4.2) | 2.8 (1.9 - 4.2) | 3.1 (2.0 - 4.7) |
| Moderate |  | 1.7 (1.1 - 2.7) | 1.6 (1.0 - 2.5) | 1.8 (1.1 - 2.8) |
| Strong/very strong |  | 1.9 (1.3 - 2.8) | 1.6 (1.0 - 2.5) | 1.7 (1.1 - 2.6) |
|  |  |  |  |  |
|  |  | **Prescription painkillers (n=69)** | | |
| No pain (reference) |  | 1 | 1 | 1 |
| Very mild/mild |  | 4.5 (2.2 - 9.2) | 4.6 (2.2 - 9.4) | 4.6 (2.2 - 9.5) |
| Moderate |  | 5.9 (3.5 - 10.1) | 5.8 (3.3 - 10.0) | 5.6 (3.2 - 10.0) |
| Strong/very strong |  | 7.0 (4.3 - 11.3) | 6.6 (3.9 - 11.3) | 6.7 (3.9 - 11.3) |
|  |  |  |  |  |
| n: Number with the given outcome.  ^1^Unadjusted estimates from Poisson regression with robust standard errors  ^2^ Estimates from Poisson regression with robust standard errors, adjusted for gender, age, education  ^3^ Estimates from Poisson regression with robust standard errors, adjusted for gender, age, education and trauma exposure | | | | |

| Table A1b: Associations (relative risk) between reported levels of chronic pain during the last four weeks and use of general practitioner (GP), emergency room services (ER), outpatient and/or specialist care, and being admitted to hospital during the last 12 months (N=353) | | | | |
| --- | --- | --- | --- | --- |
|  |  |  | | |
|  |  |  | **RR (95% CI)** |  |
| Chronic pain levels |  | **Model 1^1^** | **Model 2^2^** | **Model 3^3^** |
|  |  | **GP (n=300)** | | |
| No pain (reference) |  | 1 | 1 | 1 |
| Very mild/mild |  | 1.1 (0.9 - 1.3) | 1.1 (0.9 - 1.3) | 1.1 (0.9 - 1.3) |
| Moderate |  | 1.1 (1.0 - 1.2) | 1.1 (1.0 - 1.3) | 1.1 (0.9 - 1.2) |
| Strong/very strong |  | 1.1 (1.0 - 1.2) | 1.1 (1.0 - 1.3) | 1.1 (0.9 - 1.2) |
|  |  |  |  |  |
|  |  | **ER (n=63)** | | |
| No pain (reference) |  | 1 | 1 | 1 |
| Very mild/mild |  | 1.9 (0.8 - 4.2) | 1.6 (0.8 - 3.4) | 1.4 (0.7 - 3.1) |
| Moderate |  | 1.0 (0.4 - 2.4) | 0.8 (0.3 - 2.0) | 0.7 (0.3 - 1.8) |
| Strong/very strong |  | 2.9 (1.8 - 4.6) | 2.2 (1.2 - 3.8) | 2.0 (1.2 - 3.5) |
|  |  |  |  |  |
|  |  | **Outpatient/Specialist (n=55)** | | |
| No pain (reference) |  | 1 | 1 | 1 |
| Very mild/mild |  | 1.0 (0.3 - 4.0) | 1.1 (0.3 - 4.6) | 1.1 (0.3 - 4.6) |
| Moderate |  | 2.8 (1.5 - 5.3) | 3.1 (1.6 - 5.9) | 2.9 (1.5 - 5.7) |
| Strong/very strong |  | 4.0 (2.4 - 6.7) | 3.8 (2.1 - 6.9) | 3.9 (2.1 - 7.0) |
|  |  |  |  |  |
|  |  | **Hospitalization (n=57)** | | |
| No pain (reference) |  | 1 | 1 | 1 |
| Very mild/mild |  | 0.8 (0.2 - 3.3) | 0.8 (0.2 - 3.0) | 0.7 (0.2 - 2.9) |
| Moderate |  | 1.6 (0.8 - 3.4) | 1.5 (0.7 - 3.1) | 1.2 (0.5 - 2.7) |
| Strong/very strong |  | 3.1 (1.9 - 5.2) | 2.6 (1.4 - 4.7) | 2.4 (1.3 - 4.4) |
|  |  |  |  |  |
| n: Number with the given outcome  ^1^ Unadjusted estimates from Poisson regression with robust standard errors  ^2^ Estimates from Poisson regression with robust standard errors, adjusted for gender, age, education  ^3^ Estimates from Poisson regression with robust standard errors, adjusted for gender, age, education and trauma exposure | | | | |

| Table A1c: Associations between reported levels of chronic pain during the last four weeks and long-term impairment lasting at least 1 year, thereafter specified by type of impairment(s) (N=353) | | | | |
| --- | --- | --- | --- | --- |
|  |  |  | | |
|  |  |  | **RR (95% CI)** |  |
| Chronic pain levels |  | **Model 1^1^** | **Model 2^2^** | **Model 3^3^** |
|  |  | **Long-term impairment^4^ (n=100)** | | |
| No pain (reference) |  | 1 | 1 | 1 |
| Very mild/mild |  | 8.8 (6.0 - 13.1) | 9.2 (6.0 - 13.9) | 8.6 (5.6 - 13.4) |
| Moderate |  | 7.6 (5.1 - 11.5) | 7.6 (5.0 - 11.6) | 6.7 (4.3 - 10.5) |
| Strong/very strong |  | 7.1 (4.7 - 10.7) | 6.8 (4.4 - 10.6) | 6.7 (4.3 - 10.4) |
|  |  |  |  |  |
|  |  | **Motor ability impairment (n=81)** | | |
| No pain (reference) |  | 1 | 1 | 1 |
| Very mild/mild |  | 12.7 (7.5 - 21.5) | 13.4 (7.9 - 22.8) | 12.8 (7.4 - 22.3) |
| Moderate |  | 9.6 (5.6 - 16.7) | 9.7 (5.6 - 17.1) | 8.7 (4.7 - 15.8) |
| Strong/very strong |  | 11.1 (6.6 - 18.6) | 10.9 (6.3 - 18.9) | 10.7 (6.1 - 18.7) |
|  |  |  |  |  |
|  |  | **Vision impairment (n=24)** | | |
| No pain (reference) |  | 1 | 1 | 1 |
| Very mild/mild |  | 1.7 (0.2 - 12.9) | 1.6 (0.2 - 11.6) | 1.3 (0.2 - 7.5) |
| Moderate |  | 2.8 (0.8 - 9.9) | 2.5 (0.7 - 8.7) | 1.1 (0.3 - 5.0) |
| Strong/very strong |  | 9.2 (4.0 - 21.1) | 7.2 (2.7 - 19.0) | 6.3 (2.5 - 16.2) |
|  |  |  |  |  |
|  |  | **Hearing impairment (n=18)** | | |
| No pain (reference) |  | 1 | 1 | 1 |
| Very mild/mild |  | 4.5 (1.0 - 20.9) | 5.1 (1.1 - 23.0) | 4.7 (1.1 - 20.1) |
| Moderate |  | 3.7 (1.0 - 14.1) | 3.6 (1.0 - 13.2) | 2.0 (0.4 - 10.3) |
| Strong/very strong |  | 7.1 (2.5 - 20.2) | 5.7 (1.6 - 20.8) | 6.0 (1.8 - 19.7) |
|  |  |  |  |  |
|  |  | **Impairment due to physical illness (n=48)** | | |
| No pain (reference) |  | 1 | 1 | 1 |
| Very mild/mild |  | 12.0 (5.2 - 27.6) | 12.0 (5.2 - 27.7) | 9.9 (4.1 - 24.1) |
| Moderate |  | 10.6 (4.9 - 23.0) | 10.7 (4.7 - 24.3) | 8.5 (3.4 - 21.3) |
| Strong/very strong |  | 12.2 (5.9 - 25.4) | 11.2 (4.8 - 26.0) | 9.9 (4.2 - 23.3) |
|  |  |  |  |  |
|  |  | **Impairment due to mental illness (n=17)** | | |
| No pain (reference) |  | 1 | 1 | 1 |
| Very mild/mild |  | 9.0 (1.6 - 50.9) | 14.1 (2.2 - 90.5) | 11.2 (1.6 - 79.8) |
| Moderate |  | 2.4 (0.3 - 23.0) | 3.3 (0.3 - 33.8) | 2.5 (0.2 - 26.6) |
| Strong/very strong |  | 22.4 (6.5 - 77.2) | 27.6 (5.5 - 137.7) | 24.9 (5.0 - 123.9) |
|  |  |  |  |  |
| n: Number with the given outcome.  ^1^ Unadjusted estimates from Poisson regression with robust standard errors  ^2^ Estimates from Poisson regression with robust standard errors, adjusted for gender, age, education  ^3^ Estimates from Poisson regression with robust standard errors, adjusted for gender, age, education and trauma exposure  ^4^ Defined as long-term (at least 1 year) illness or injury of a physical or psychological nature that impairs daily life | | | | |
